# Oncologic oUTcomes of neoadjuvant chemotherapy for obSTructive colon cAncer after steNt decompression (OUTSTAND trial); A study protocol of multicenter non-inferiority randomized controlled trial

**DOI:** 10.1186/s12885-025-13588-0

**Published:** 2025-02-03

**Authors:** Bong-Hyeon Kye, Ji-Hoon Kim, Hyung-Jin Kim, Yoon-Suk Lee, In-Kyu Lee, Won Kyung Kang, Hyeon-Min Cho, Jong-Kyung Park, Chang-Hyeok Ahn, Jae-Im Lee, Seong-Taek Oh, Byung Jo Choi

**Affiliations:** 1 Department of Surgery, St. Vincent’s Hospital, College of Medicine, The Catholic University of Korea, Suwon, Korea; 2 Department of Surgery, Incheon St. Mary’s Hospital, College of Medicine, The Catholic University of Korea, Incheon, Korea; 3 Department of Surgery, Eunpyeong St. Mary’s Hospital, College of Medicine, The Catholic University of Korea, Seoul, Korea; 4 Department of Surgery, Seoul St. Mary’s Hospital, College of Medicine, The Catholic University of Korea, 505 Banpo-dong, Seocho-gu, Seoul, 06591 Korea; 5 Department of Surgery, Yeouido St. Mary’s Hospital, College of Medicine, The Catholic University of Korea, Seoul, Korea; 6 Department of Surgery, Bucheon St. Mary’s Hospital, College of Medicine, The Catholic University of Korea, Bucheon, Korea; 7 Department of Surgery, Uijeongbu St. Mary’s Hospital, College of Medicine, The Catholic University of Korea, Uijeongbu, Korea; 8 Department of Surgery, Daejeon St. Mary’s Hospital, College of Medicine, The Catholic University of Korea, Daejeon, Korea

**Keywords:** Colon cancer, Colon obstruction, Self expandable metallic stent, Neoadjuvant chemotherapy, Bridge to surgery

## Abstract

**Background:**

For obstructive colon cancer, many studies have been conducted on the use of self-expandable metallic stents (SEMS) as a bridge to surgery (BTS). However, there are currently no available prospective data on the impact of bridging period and there is a lack of research on the effects of neoadjuvant chemotherapy during the bridging period.

**Objectives:**

Patients who undergo successful SEMS placement for obstructive left-sided colon adenocarcinoma without metastases will be eligible for this study.

**Design:**

This study is a multicenter, non-inferiority, randomized (1:1), open-label, controlled trial.

**Methods & analysis:**

The patients assigned to the control group will undergo curative surgery within two weeks after successful SEMS placement. The patients assigned to the experimental group will undergo three cycles of neoadjuvant FOLFOX chemotherapy within two weeks after successful SEMS placement. Curative surgery will be performed within four weeks of the last administration of neoadjuvant FOLFOX. Circulating tumor DNA (ctDNA) will be collected at specific time points.

**Discussion:**

The optimal time interval for SEMS placement as a BTS can significantly impact long-term oncologic outcomes. In this study, our goal is to identify the optimal time interval for SEMS placement as a BTS. Recently, there has been interest in applying neoadjuvant chemotherapy for locally advanced colon cancer. In the context of early treatment for tumor dissemination following SEMS placement, neoadjuvant chemotherapy may be beneficial for delayed surgery after SEMS placement as a BTS. The results of this trial will be an important reference for the application of neoadjuvant chemotherapy in locally advanced colon cancer. Additionally, researchers will investigate whether ctDNA can serve as a reliable indicator to guide decisions about the timing and type of subsequent treatment. Based on the results of this trial, a patient-tailored treatment strategy can be developed for obstructive colon cancer.

**Registration:**

This study is registered on ClinicalTrials.gov Identifier: NCT04889820, registered on May 17, 2021 in clinicaltrials.gov; Protocol ID: XC21MIDI0004.

**Supplementary Information:**

The online version contains supplementary material available at 10.1186/s12885-025-13588-0.

## Introduction

It is known that around 30–40% of colorectal cancer require emergency surgery. Colonic obstruction accounts for 80% of emergency treatment cases, while colon perforation accounts for the remaining 20% [1–3]. Patients with colorectal cancer who require emergency surgery have higher rates of postoperative complications, mortality, and ostomy formation compared to those who do not require emergency surgery [4, 5]. Patients who undergo emergency surgery tend to have worse long-term survival rates in terms of oncological outcomes [6]. Despite recent efforts to actively screen for colorectal cancer, some studies have reported that the proportion of cases resulting in obstruction or perforation requiring emergency surgery has remained unchanged [7, 8]. However, research on treatments aimed at reducing postoperative complications and improving long-term survival for patients with symptomatic colorectal cancer who require emergency surgery is still insufficient.

Emergency surgery cannot be avoided when colorectal cancer causes perforation of the colon. Obstructive colorectal cancer has traditionally been treated with emergency surgery, which involves resecting the affected part of the colon and creating a stoma. With the advancements in endoscopic equipment and technology, it has become possible to insert endoscopic stents, such as self-expandable metallic stents (SEMS), in patients with obstructive colon cancer. Numerous studies have been conducted on the use of SEMS as a bridge to surgery (BTS), which involves performing surgery after achieving sufficient decompression and bowel lavage through the placement of SEMS [9]. These studies have shown that a BTS can reduce postoperative complications, stoma formation, and postoperative mortality in select cases [9]. Currently, these studies recommend the use of SEMS to improve short-term postoperative outcomes. However, the long-term oncological outcomes of SEMs as a BTS have not yet been established.

After SEMS placement, the primary objectives of BTS may include performing oncologic surgery on patients with obstructive colon cancer who are in a more stable or improved physical condition, conducting one-stage surgery to avoid the need for a diverting stoma, and reducing postoperative morbidity. To achieve the dual goals of improved perioperative and oncologic outcomes, some studies have focused on optimizing the interval between the placement of SEMS and elective surgery [10, 11]. The current guideline from the European Society of Gastrointestinal Endoscopy (ESGE) suggests a time interval of approximately two weeks before resection when colonic stenting is used as a bridge to elective surgery for patients with curable left-sided colon cancer [12]. However, there are currently no available prospective comparative data on the impact of this period on surgery, complications, or overall and disease-free survival. Furthermore, there is a lack of significant research on the effects of neoadjuvant chemotherapy during the bridging period.

In this study, our objective is to determine the optimal time interval for curative resection after successful placement of SEMS for obstructive colon cancer. Additionally, our aim is to investigate whether neoadjuvant chemotherapy, followed by curative resection, can improve short-term perioperative outcomes and long-term oncological outcomes.

## Methods and design

### Patient enrollment

This study was designed as a multicenter, prospective, non-inferiority, randomized, controlled comparative study. The study will last approximately six years, consisting of three years of inclusion and three years of follow-up. Patients will be enrolled at eight University hospitals affiliated with The Catholic University of Korea, including Seoul St. Mary’s Hospital, Yeouido St. Mary’s Hospital, Uijeongbu St. Mary’s Hospital, St. Vincent’s Hospital, Incheon St. Mary’s Hospital, Eunpyeong St. Mary’s Hospital, Bucheon St. Mary’s Hospital, and Daejeon St. Mary’s Hospital. Complete information will be provided to patients and their guardians after the successful placement of SEMS for obstructive lesions on the left side of the colon. Informed consent will be obtained from the patients after pathologic confirmation of adenocarcinoma and completion of preoperative staging work-up.

Consecutive adult patients between the ages of 20 and 75 with pathologically confirmed adenocarcinoma will be eligible if they meet the following criteria: clinical stage II or III colon cancer with colonic obstruction; a colon cancer located between the splenic flexure colon and rectosigmoid junction colon, defined as a tumor located above 15 cm from the anal verge; no colonic injury and successful decompression of the colon within 48 h after SEMS placement; Eastern Cooperative Oncology Group (ECOG) performance status of 0–2; American Society of Anesthesiologists (ASA) physical status grade I, II, or III; and a negative urine human chorionic gonadotropin (hCG) test in women who are in their fertile period.

Patients will be excluded from the study if there is any suspicion of distant metastasis or the presence of another primary malignant lesion. Additionally, patients with a clinical stage of T1 or T2 and N0 will be excluded if they exhibit signs of perforation or severe ischemia that necessitates emergency surgery. Other exclusion criteria include complications related to the placement of self-expanding metal stents (SEMS), such as infection, severe bleeding, or perforation following SEMS placement, as well as colonic obstruction caused by benign strictures.

### Ethics

We have already obtained approval from the review board of The Catholic University of Korea, CMC Clinical Research Coordination Center (XC21MIDI0004). The patients will be enrolled in the study, and their clinical information will be collected using a predetermined dataset. The informed consent will be obtained after confirming the successful placement of SEMS, in accordance with the guidelines and regulations for our institution’s prospective randomized controlled study. The review board will handle adverse events, and researchers will report any related to the study protocol. This study has also been registered on ClinicalTrials.gov with the identifier NCT04889820. This is version 1.0 for this study.

### Definition

In this study, left-sided obstructive colon cancer will be defined as a confirmed obstructive lesion caused by adenocarcinoma, which arises from the splenic flexure colon to the rectosigmoid colon. Obstructive colon cancer will be diagnosed when patients complain of symptoms such as abdominal pain, distension, and the absence of stool and flatus passage. Radiologic findings from an abdomen and pelvic computed tomography (CT) may reveal severe dilation of the proximal colon due to a suspicious obstructive lesion. Patients exhibiting any signs of generalized peritonitis due to colonic perforation will be excluded from the study on obstructive colon cancer.

### SEMS insertion and preoperative preparation

In patients with abdominal pain and distension, we will initially conduct a plain X-ray. If colon obstruction is suspected, we will then proceed with an abdominopelvic CT scan. If radiologists suspect obstruction from colon cancer, we will explain to the patient and their guardians the need and risks associated with colonoscopic biopsy and stent placement. Once informed consent is obtained, all SEMS procedures will be performed by experienced gastroenterologists within 48 h of the initial hospital visit at each hospital that is equipped with SEMS. The SEMS will be inserted by a gastroenterologist with the aid of colonoscopic and/or fluoroscopic guidance at all hospitals. The HANARO stent (M.I. Tech Co., Ltd, Seoul, South Korea) or the Niti-S stent (Taewoong Medical, Co., Ltd, Gyeonggido, South Korea) will be used in all cases. After successfully inserting the SEMS, complications, expansion of the SEMS, and resolution of the intestinal obstruction will be monitored through serial plain abdominal films. Patients will undergo chest CT or positron emission tomography-CT (PET-CT) scans, and serum carcinoembryonic antigen (CEA) levels will be obtained after confirmation of adenocarcinoma through colonoscopic biopsy. Additionally, a colonoscopy will be performed after mechanical bowel preparation to identify any other colonic neoplasms once the SEMS has fully expanded and the patient’s abdominal symptoms have disappeared. Before randomization, a full colonoscopic evaluation will be performed to identify any lesions in the proximal colon. If there is no colon cancer in the proximal region, patients will be eligible to participate in this study. However, when a full colonoscopic evaluation is difficult to perform for various reasons, evaluating the proximal colon through PET-CT can be considered as an alternative option. All patients will receive perioperative intravenous antibiotics, and mechanical bowel preparation will be performed prior to surgery. After obtaining informed consent, the patients will be enrolled and randomly assigned to either the control group or the experimental group.

### The control group

The patients assigned to the control group will undergo curative surgery within two weeks after successful SEMS placement. After their recovery from surgery, they will receive adjuvant FOLFOX chemotherapy within four weeks. Adjuvant FOLFOX chemotherapy will be administered every two weeks for six months, totaling 12 cycles.

### The experimental group

**Fig. 1:**
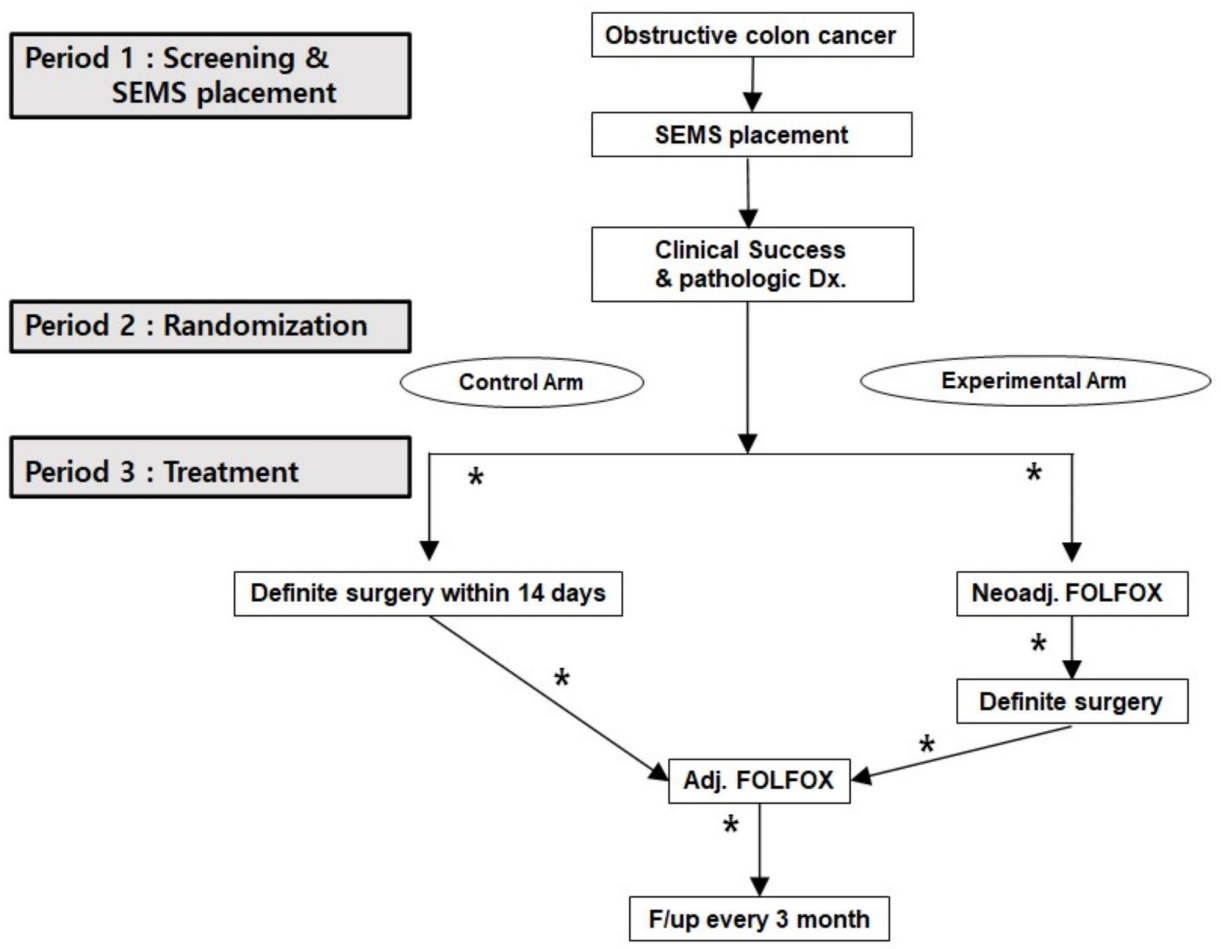
The flowchart of the study on screening and treatment

The patients assigned to the experimental group will undergo neoadjuvant FOLFOX chemotherapy within two weeks after successful SEMS placement. After three cycles of neoadjuvant FOLFOX chemotherapy, the patient will undergo a follow-up study, including a serum CEA, abdomen and pelvic CT, and either a chest PA or chest CT, to determine the status of the tumor. If no metastatic lesions are found in these assessments, curative surgery will be performed within four weeks of the last administration of FOLFOX. After their recovery from surgery, they will receive adjuvant FOLFOX chemotherapy within four weeks. Adjuvant FOLFOX chemotherapy will be administered every two weeks for approximately four months, totaling nine cycles. The experimental group will receive a total of 12 cycles of perioperative FOLFOX chemotherapy. The flowchart of the study is presented in Fig. 1.

### Chemotherapy

Two different regimens of oxaliplatin with 5-fluorouracil/leucovorin (FOLFOX) will be allowed in this study protocol. There are two types of chemotherapy regimens: FOLFOX-4 and mFOLFOX-6. FOLFOX-4 involves administering Oxaliplatin at a dose of 85 mg/m^2^ intravenously (IV) on the first day in 500 cc of 5% dextrose water over 2 hours, along with leucovorin at a dose of 400 mg/m^2^ IV or levoleucovorin at a dose of 200 mg/m^2^ IV. Additionally, 5-FU is administered at a dose of 400 mg/m^2^ as an intravenous bolus, followed by a continuous infusion of 600 mg/m^2^ for 22 h on the first and second days. The mFOLFOX-6 regimen involves administering Oxaliplatin at a dose of 85 mg/m^2^ IV on the first day in 500 cc of 5% dextrose water over 2 hours. Additionally, leucovorin is given at a dose of 400 mg/m^2^ IV or levoleucovorin at a dose of 200 mg/m^2^ IV. Additionally, 5-FU is administered at a dose of 400 mg/m^2^ IV bolus, followed by a continuous infusion of 2,400 mg/m^2^ IV over 46 h on the first and second days. The cycle will be repeated every two weeks in both regimens.

### Sample size and randomization

Based on our retrospective analysis, the 5-year overall survival (OS) rate for the control group was 84.1%. However, for patients who underwent curative surgery 4 weeks after SEMS placement without neoadjuvant chemotherapy, the OS rate was only 37.5%. The maximum response rate was assumed to be 80.0%, while the minimum response rate was assumed to be 40.0%. The non-inferiority limit for this study was set at 20%, which is half of 40%. Additionally, a non-inferiority margin of 60% was established. We determined the noninferiority margin for our study based on clinical judgment and retrospective data. We have chosen the noninferiority test as a method to minimize the unrealistic overall survival difference resulting from such a significant gap. We have set the non-inferiority limit for this study at 20%, which is relatively large. However, we believed that a 20% increase in OS with neoadjuvant therapy is possible and realistic, even though it is not a small number. Although it is generally advisable to choose a smaller value for the noninferiority margin, the feasibility of study recruitment was another important factor to consider. Therefore, we selected the noninferiority margin equivalent to a 20% increase in the expected OS rate. A non-inferiority log-rank test was conducted with a total sample size of 204 subjects, evenly divided between the control and experimental groups. The test achieved 80.1% power at a significance level of 0.050 to detect an equivalence hazard ratio of 2.29, assuming the actual hazard ratio is 1.00 and the hazard rate of the reference group is 0.0446. The study will last for six years, during which subject accrual (entry) will occur within the first three years. The accrual pattern is uniform across all time periods; all years are equal. The proportion of participants dropping out of the control group is 0.0100. The proportion of participants dropping out of the experimental group is 0.0100. The proportion of individuals switching from the control group to another group with a hazard rate equal to that of the experimental group is 0.0000. The proportion of individuals switching from the experimental group to another group with a hazard rate equal to that of the reference group is 0.0000. The estimated failure rate for stents is 8%. And, the failure rate for screening - indicating synchronous malignancies at other sites or patients requiring emergency operations - is 15%. A total of over 260 patients will be screened for the successful trial.

After the successful placement of self-expandable metal stents (SEMS), random allocation will be carried out with the consent of patients and their guardians. A total of 204 patients will be evenly distributed between the two groups in a 1:1 ratio. This study is a multicenter, randomized (1:1), parallel-group, open-label, controlled clinical trial. Using a random-number table, assignment codes will be concealed in opaque envelopes. To avoid the issue of tampering with the allocation from the sealed envelopes, the sealed opaque envelopes containing the randomization card, which is not visible through transillumination, are stored at the trial administration center (St. Vincent’s Hospital, Suwon, Korea). They are opened by a third party immediately after receiving a telephone call from the clinician who wishes to enroll a patient, in order to minimize the risk of allocation manipulation. The sealed envelopes will be used but will be open by a centralized system. Half of the patients will be randomly assigned to the control group, while the other half will be assigned to the experimental group through randomization.

### Follow-up

Follow-up data will be obtained for all patients during routine clinical practice. During each follow-up office visit, patients will undergo evaluation using CEA, abdomen and pelvic CT, and either chest PA or chest CT. Annual colonoscopic surveillance will be performed. Patients will undergo examinations every three months for the first two years, and then every six months for the remaining three to five years of the schedule.

### Endpoint

The primary endpoint will be the 3-year OS rate after curative surgery. The time of SEMS placement will serve as the origin for survival analysis in both groups, ensuring a consistent starting point. The rates of complications related to SEMS, postoperative complications within 30 days after surgery, chemotherapy-related adverse events, stoma formation, stoma-free survival, recurrence, 3-year disease-free survival (DFS), and QoL will be analyzed as secondary outcomes. Circulating tumor DNA (ctDNA) will be collected at specific time points, including after SEMS placement, before and after curative resection, after neoadjuvant chemotherapy, and after every three cycles of chemotherapy. Through the analysis of serial samples of ctDNA, our objective is to determine the correlation between treatment strategies and oncologic outcomes. This will provide a basis for individualizing treatment in patients with obstructive colon cancer.

### Circulating tumor DNA analysis

Peripheral venous blood samples will be collected from patients at the aforementioned time points. At least 20 mL of blood will be collected in tubes containing EDTA. The plasma was separated within four hours of sample collection. The plasma obtained will undergo centrifugation at 2000 g for 5 min and at 16,000 g for 10 min. It will then be immediately aliquoted and stored at -80 °C. To isolate cell-free DNA from 4 mL of plasma, we will use a MagMAX Cell-Free DNA Isolation Kit (Applied Biosystems, Foster City, CA, USA) along with a KingFisher Duo Prime Magnetic Particle Processor (Thermo Fisher Scientific, Waltham, MA, USA), following the instructions provided by each manufacturer. The concentration of purified plasma cell-free DNA was measured using a Qubit 2.0 fluorometer (Thermo Fisher Scientific, Waltham, MA, USA) in conjunction with a Qubit dsDNA HS Assay Kit (Thermo Fisher Scientific, Waltham, MA, USA), following the manufacturer’s instructions.

The sequence data will undergo primary and secondary analyses using the standard Ion Torrent Suite Software, which will be operated on a Torrent Server. The raw signal data will be analyzed using Torrent Suite v5.10.1 and Ion Reporter software (Thermo Fisher Scientific, Waltham, MA, USA). The pipeline will include several steps, such as signal processing, base calling, quality score assignment, adapter trimming, PCR duplicate removal, read alignment, quality control of mapping quality, coverage analysis, and variant calling. The sequencing reads will be aligned to the UCSC hg19 reference genome (Genome Reference Consortium GRCh37). Sequence variants will be identified using the Ion Reporter software v5.10 and the Ion AmpliSeq HD Workflow template for Liquid Biopsy - w1.4 - DNA - Single Sample (Thermo Fisher Scientific, Waltham, MA, USA). The coverage of each amplicon will be determined using the Coverage Analysis Plugin software version 5.10.0 (Thermo Fisher Scientific, Waltham, MA, USA). The use of UMIs will enable the categorization of reads into molecular families. Random errors generated during the library construction and sequencing process will be automatically removed.

### Statisticl analysis

The analysis will be conducted following the intention-to-treat (ITT) principle, meaning that patients will be evaluated based on their assigned treatment, regardless of whether they completed the adjuvant chemotherapy. Additionally, a per-protocol (PP) analysis will be performed, which will consider the treatment that was actually administered, except in cases of significant deviations, such as early dropout before follow-up evaluations or during treatment. The primary analysis will encompass both ITT and PP populations. For the non-inferiority assessment of the primary endpoint, a one-sided 95% confidence interval (CI) will be calculated using the unadjusted Cox proportional hazards model. Non-inferiority will be declared if the upper limit of this one-sided 95% CI of the hazard ratio (HR) is less than 2.29 (the pre-specified non-inferiority margin) in both the PP and ITT analyses. The proportionality assumption will be assessed using both log-negative-log plots and Schoenfeld residuals. If it is determined that the assumption of proportional hazards has been violated, a landmark analysis will be performed by defining a clinically meaningful time point. Comparisons between continuous variables will be conducted using the Wilcoxon rank-sum test or the two-sample t-test, depending on the data’s normality. Categorical variables will be analyzed using the chi-square test. Survival probability analysis will be conducted using the Kaplan-Meier method. The log-rank test will be used to assess the difference in survival rates between groups. Significance will be defined as a p-value of 0.05 or lower. All statistical analyses will be performed using version 23.0 of the Statistical Package for the Social Sciences (SPSS) for Windows (SPSS, Inc., Chicago, IL).

### Study organization

The responsibility for conducting the trial lies with St. Vincent’s Hospital in Suwon, Korea. The study was carried out by the Division of Colorectal Disease in the Department of Surgery at The Catholic University of Korea in Seoul, Korea. The division is composed of surgeons who specialize in colorectal surgery in Korea. To ensure the study procedures are harmonized and the progress is monitored and shared, periodic board meetings will be scheduled approximately every three months.

### Dissemination

Results of the study will be presented at local, national and international medical meetings. The findings of the study will be published in peer reviewed medical/scientific journals and made open access on acceptance. Information may also be disseminated to the general public via public engagement and community outreach programmes.

## Discussion

Currently, ESGE recommends the placement of self-expandable metal stents (SEMS) as a bridge to surgery (BTS) for patients with left-sided obstructing colon cancer that is potentially curable [12]. This is considered an alternative to emergency surgery. This recommendation is based on the favorable short-term results of using SEMS placement as a BTS compared to emergency surgery, as well as the similar long-term oncologic outcomes between SEMS placement as a BTS and emergency surgery [12–15]. Colonic obstruction can cause edema in the proximal colon, resulting in a decline in the general condition of patients. This can lead to a range of serious complications, including imbalances in water and electrolytes, acid-base imbalances, peritonitis, intestinal perforation, and septic shock [10]. Although emergency surgery can relieve obstruction symptoms, it may also result in severe postoperative morbidity and mortality due to the unstable condition of the patients. Most studies related to colon cancer obstruction have demonstrated lower rates of permanent stomas and higher rates of primary anastomosis with the use of SEMS as a BTS compared to emergency surgery [14–16]. Until recently, SEMS were not considered as an alternative treatment option for colon cancer obstruction due to various features after SEMS placement, such as tumor dissemination and silent perforation. Based on several meta-analyses of DFS and OS, recent clinical data suggest that SEMS placement is a beneficial treatment option for colon cancer obstruction. It appears that SMES placement as a BTS can be a recommended treatment option for colon cancer obstruction [12, 13, 15, 16]. Nonetheless, the long-term oncologic safety of using SEMS placement as a BTS requires further study and clarification.

The optimal time interval for SEMS placement as a BTS can significantly impact long-term oncologic outcomes. After SEMS placement, the primary objectives of BTS may include performing oncologic surgery on patients with obstructive colon cancer in a more stable or improved physical condition, conducting one-stage surgery to avoid the need for a diverting stoma, and reducing postoperative morbidity [10, 11]. To achieve this, it is necessary to establish the optimal time interval between SEMS placement and elective surgery. However, there is limited data available regarding the optimal time interval. One strategy could be to schedule elective surgery as soon as possible after SEMS placement to reduce the risk of tumor dissemination and alteration of pathological findings. This approach can also minimize the interaction between the tumor and the prosthesis, such as a stent. A retrospective study conducted in Japan with 47 patients who underwent BTS after SEMS placement demonstrated that the interval of 15 days between SEMS placement and surgery was the only independent risk factor for postoperative complications. They recommended an interval of more than 15 days to minimize postoperative complications [17]. In an Italian study, the authors found that various time thresholds did not have a correlation with the occurrence of postoperative morbidity [18]. However, the ROC curve for postoperative morbidity indicated that waiting for at least six days could be appropriate surgical timing. A multicenter retrospective study conducted in Denmark revealed that the risk of recurrence significantly increased in the group with a time interval of more than 18 days [19]. By using an “intention-to-treat” model that included patients who underwent emergency surgery due to complications from stent placement, the researchers found that the risk of recurrence was significantly higher in the group with a time interval of more than 18 days. Our multicenter retrospective study found that the time interval between SEMS placement and elective surgery did not significantly affect perioperative short-term outcomes [10]. However, early elective surgery within 7 days, or at least within 14 days, after SEMS placement may reduce the oncologic risk of BTS. In this study, we aim to analyze the short-term perioperative outcomes and long-term oncologic outcomes based on the time interval between SEMS placement and elective surgery. Our goal is to identify the optimal time interval for SEMS placement as a BTS.

Recently, there has been interest in applying neoadjuvant chemotherapy for locally advanced colon cancer [20–22]. Theoretical advantages of neoadjuvant chemotherapy include early treatment of potential lymph node and/or distant micrometastases, an increased likelihood of achieving a clear resection margin, and the ability to evaluate chemosensitivity and assess tumor biology based on the degree of downstaging that may occur after treatment. In the context of early treatment for tumor dissemination following SEMS placement, neoadjuvant chemotherapy may be beneficial for delayed surgery after SEMS placement as a BTS [23, 24]. 

Circulating cell-free DNA (cfDNA) is derived and released from apoptotic or necrotic cells, and circulating tumor DNA (ctDNA) with tumor-specific DNA from tumor cells undergoing apoptosis or necrosis is released into the systemic circulation. The measurement of ctDNA has been suggested its clinical application in screening, diagnosis, and predicting tumor response or resistance to treatment [25]. During SEMS placement for obstructive colon cancer, manipulation of the tumor during colonoscopy, increased interstitial pressure, and clinical or silent perforation can lead to the dissemination of cancer cells into the peripheral circulation. Moreover, the use of SEMS can lead to the dissemination of tumor cells due to mechanical compression of the guidewire and air insufflation, which violates the fundamental principle of oncologic treatment [26]. In other words, SEMS placement for obstructive colon cancer can have an impact on the tumor microenvironment. Additionally, ctDNA with tumor-specific DNA can be released into the peripheral circulation after SEMS placement. The measurement of ctDNA with tumor-specific DNA may help explain the oncologic risk associated with SEMS placement. This information can assist in making a more informed decision regarding the use of SEMS for obstructive colon cancer. In this study, ctDNA will be collected at regular intervals and analyzed to assess its potential as a tool for monitoring therapeutic efficacy. Specifically, researchers will investigate whether ctDNA can serve as a reliable indicator to guide decisions about the timing and type of subsequent treatment.

To the best of our knowledge, this study will be the first prospective trial to evaluate the oncologic safety of neoadjuvant chemotherapy for obstructive colon cancer following successful SEMS placement. The results of this trial will be an important reference for the application of neoadjuvant chemotherapy in locally advanced colon cancer. The results of ctDNA testing may aid in the decision-making process for selecting emergency surgery or SEMS placement, regardless of whether the patient has received neoadjuvant chemotherapy, in cases of obstructive colon cancer. Based on the results of this trial, a patient-tailored treatment strategy can be developed for obstructive colon cancer.

## Electronic supplementary material

Below is the link to the electronic supplementary material.

Supplementary Material 1

## Data Availability

No datasets were generated or analysed during the current study.
